# Association between Dietary Patterns and Major Depression in Adult Females: A Case-Control Study

**DOI:** 10.34172/jrhs.2021.37

**Published:** 2021-01-12

**Authors:** Sakineh Nouri Saeidlou, Arezou Kiani, Parvin Ayremlou

**Affiliations:** ^1^Social Determinants of Health Research Center, Urmia University of Medical Sciences, Urmia, Iran; ^2^Clinical Research Development Unit of Imam Khomeini Hospital, Urmia University of Medical Sciences, Urmia, Iran

**Keywords:** Dietary Patterns, Food Security, Major Depression

## Abstract

**Background:** Depression is one of the most common mental disorders. This study aimed to determine the association between dietary patterns and major depression in adult females.

**Study design:** A case-control study.

**Methods:** This study was conducted on adult females suffering from major depression within the age range from 19 to 65 years. The total participants of this study included 170 cases and 340 controls. Dietary intakes were collected using a 168-item validated semi-quantitative food-frequency questionnaire. Household food security was measured using a locally adapted Household Food Insecurity Access Scale. Moreover, the depression status of the adult females was assessed through a validated "Beck" questionnaire. Logistic regression was utilized to assess the association between dietary pattern scores and depression.

**Results:** The mean ±SD ages of the participants were 36.97 ±11.28 and 36.07 ±10.58 years in the case and control groups, respectively (*P*=0.374), and five major dietary patterns were extracted in this study. The odds ratio (OR) in the last adjusted model was (OR: 0.61; 95% CI: 0.46, 0.81); therefore, the "Healthy pattern" was significantly inversely associated with the odds of depression. Adherence to the "Western pattern" significantly increased depression by 29% (OR: 1.29; 95% CI: 1.06, 1.59). Furthermore, the "Traditional pattern" was positively associated with depression (OR: 1.16; 95% CI: 0.94, 1.43). There was no significant association between "Sugar and fast food" and "red meat and oils" dietary pattern and depression.

**Conclusions:** Healthy dietary pattern reduces the risk of depression in adult females; however, the western and traditional dietary patterns increases this risk.

## Introduction

Depression disorders are major public health concerns for developed and developing countries^1-3^. According to the World Health Organization report, depression is the second-largest cause for the burden of disease in 2020 ^4^.

Globally, depressive disorders affect 350 million people in all age groups, and they are more prevalent in adult females ^5,6^. According to a national study conducted in Iran, depression was the third health problem in this country ^2^. Furthermore, the studies showed that depression was higher in adult females (about 1.7 times higher), compared to males ^7,8^. In Iran, major depression is the most prevalent mood disorder (2.98 % in total, 4.38 % in adult females) ^9^.

According to a high prevalence of depression and the significant economic burden resulted from this disease, it is necessary to assess the associated risk factors, such as diet. Recently, many epidemiological studies showed that diet, as a modifiable factor was related to oxidative stress, inflammation, as well as brain plasticity and function. All of these factors play an important role in the mental health of the person and the development of depression ^10^. A separate examination of the nutrients or foods in a diet and mental health does not provide a complete picture of the relationship due to the complex interplay of nutrients in the diet. Therefore, the analysis of food patterns as a Community Approach has attracted much attention from researchers ^11-13^.

Major depression is a type of mood disorder that includes a variety of symptoms, such as sadness, lack of pleasure, feeling depressed, absence of interest or enjoyment, too little or too much sleep, increased or decreased appetite, together with weight changes, restlessness, guilt or worthlessness, low energy, difficulty in concentration, as well as attitudes toward worthlessness of life and suicidal behaviors. To confirm the diagnosis of major depression, at least five of these symptoms should be present in an individual for two weeks ^11,12^.

 The previous studies have mainly investigated the association between the diseases and lack or presence of single or multiple nutrients or foods. This type of analysis has several conceptual and methodological limitations. The individual does not use a nutrient alone, rather it is used in the form of food composition. Therefore, it is very hard to measure the effect of a single nutrient, while it is quite easy to measure the collective effects of some nutrients in a dietary pattern. It should be noted that the identification of the relationship between depression and a specific nutrient is problematic due to the probability of synergistic effects or interventions in a diet.

Due to the lack of sufficient information on the association between dietary patterns and depression, along with the high prevalence of depression in adult females ^13^, the present study was conducted to determine the relationship between dietary patterns and major depression in adult females.

## Methods

###  Study participants

 This unmatched case-control study was conducted on new cases of major depressive disorder in adult females from May 2018 to February 2018. The participants included 170 cases and 340 controls within the age range from 19 to 65 years who were referred to the referral psychiatric clinics in Urmia, Iran. A psychiatrist diagnosed the major depression in the cases based on the clinical examination and a standard Beck Depression Inventory. According to the Beck Inventory, a score equal to or more than 36 indicated major depressive disorder. The inclusion criteria were: 1) adult females, 2) age range from 19 to 65 years, and 3) recently diagnosed with major depression (score>36 based on Beck questionnaire) diagnosed by a psychiatrist. Moreover, the controls were those who obtained a score between 1-18 based on the Beck questionnaire with no history of depression in the past year.

 On the other hand, the adult females with cognitive impairments or other psychotic and hormonal disorders, chronic illnesses, and a history of alcohol or drug addiction, as well as pregnant or lactating females or those with such high degree of depression in which they were unable to answer any questions were excluded from the study. Moreover, the females who lost a family member in the past six months and were following a specific diet in the past year were not included in the study.

The study protocol was approved by the Ethical Committee of Urmia University of Medical Sciences, Urmia, Iran (ID: IR.UMSU.REC.1397.184). Regarding the ethical considerations, informed consent was obtained from all participants. The sample size was calculated based on the appropriate formulas, odds ratio (OR) of 3.619 for an unhealthy dietary pattern in a previous study (3) with a prevalence of major depression (4.38%) in adult females^14^, 95% CI (Z1-α2=1.96), power of 90% (Z1-β=1.28), control:case ratio of 2:1, and 20% additional samples. Eventually, 170 and 340 cases and controls were included in this study, respectively.

###  Dietary intake assessment

A validated 168-item semi-quantitative food-frequency questionnaire (FFQ) was used to collect the dietary intakes^15,16^. Participants were asked about the frequency of the consumption of each food item in a commonly used unit or portion size (daily, weekly, and monthly) over the past year. The daily intake of food items (gram/day) was calculated based on the reference book “Guides of Coefficients and Household Scales”^17^. Therefore, the amount of daily intake (gram/day) of food items was calculated through the multiplication of the portion sizes by the consumption frequency. For the calculation of the consumed food items in a week or month, the product of multiplication (portion size by consumption frequency) was divided by 7 or 30, respectively. Moreover, in addition to the above calculations, the number of months for the seasonal fruits were multiplied and divided by 365. Consequently, the FFQ food items were classified into separate food groups based on the similarity of their nutrient profile or previous studies ^3,18^. In case the food item was very different from other items (e.g., eggs, tea, and coffee), it was classified as an independent food group. Eventually, factor analysis was utilized to identify the major dietary patterns.

###  Food security status

Household food security was measured using a locally adapted Household Food Insecurity Access Scale (HFIAS), developed by Food and Nutrition Technical Assistance (FANTA) Project^19^. The scale contained nine items and, each item was given a score between 0-4. For all the items, a zero score indicated food security. Additionally, scores one, two, three, and four indicated mild, moderate, and severe food insecurity, respectively. The validity of HFIAS has been previously confirmed by the Iranian population ^20,21^. Data were collected through face-to-face interviews.

###  Beck long-form questionnaire 

The depression status of females was assessed using a validated Beck questionnaire together with interviews. The Beck Depression Inventory is a self-rating depression scale with 21 questions. Each question has four options that indicate the severity of depression from low to high. The participants read the questions in order and specify the options that best express their moods in the last two weeks. A total of 21 options determine the overall depression score that indicates Healthy Status (1-18), Mild Depression (18-28), Moderate Depression (29-35), and Severe Depression (36-63) ^22,23^.

###  Metabolic equivalent of task 

Physical activity was measured and recorded using the International Physical Activity Questionnaire (IPAQ). This questionnaire has high reliability and is significantly associated with the results of the IPAQ and 7-day records of Iranian youth physical activity^24,25^. Physical activity was assessed in minutes per week spent on common leisure-time physical activities expressed as the metabolic equivalent of task (MET)-min/week^26^.

###  Baseline characteristics and covariates

The demographic characteristics, including age, marital status, education level, family history of depression, occupational status, sleep duration (day and night), smoking, body mass index (BMI), MET, and food security were obtained via face to face interviews with participants. Moreover, the body weight was measured with light clothing (about 0.01 kg) and shoes removed using a digital scale. Height was also measured in the standing position without shoes using a strip meter with an accuracy of 0.1 cm. The BMI was calculated by the weight (kg) divided by the square of the height (m^2^).

###  Statistical analysis

Continuous and categorical variables were summarized as mean± SD, as well as number and percentages, respectively. The demographic characteristics of the cases and controls were compared using the Person’s Chi-square Test in terms of categorical variables, and the mean of continuous variables was compared using an independent t-test between the two groups. Moreover, the normality of data was tested using the Kolmogorov-Smirnov test. Principal component analysis (PCA) was conducted to identify the major dietary patterns based on the 34 food groups (Table 1). Furthermore, the components were rotated by a varimax rotation with Kaiser Normalization. The numbers of components retained were determined using a linear combination of the eigenvalues (>1.7) in the scree plot. Therefore, five factors were considered major dietary patterns and labeled based on the interpretation of the data. These five distinct factors were labeled Healthy, Sugar, and Fast foods, Western, Traditional, and Unhealthy dietary patterns. These factors explained approximately 35.8% of the total variance. Sampling adequacy and inter-correlation of factors were supported by Kaiser-Meyer-Olkin measure (0.63) and Bartlett’s test of sphericity (<0.001), respectively. Factor scores of dietary patterns for each participant were calculated through the observed intakes of the food items weighted by the factor loadings of >|0.20|, which contributed to the component and were used to name the dietary patterns. The ORs and their 95% confidence intervals (CI) were computed for assessing the association between dietary pattern scores and depression. The ORs were calculated by the logistic regression using crude and two adjusted models. In all regression models for each dietary pattern, model 1 was adjusted for marital status, education level, family history of depression, occupational status, smoking, daily sleep, and night sleep. In model 2, income and food security were also added to the model. The data were analyzed using Stata software (version 14.0). A p-value of less than 0.05 was considered statistically significant.

## Results

Table 2 tabulates the main demographic characteristics of the participants, including 170 cases and 340 controls. The variables, such as job, smoking, education level, marital status, sleep duration (day and night), income level, and family history of depression were statistically significant in both groups. However, the cases had lower education levels, compared to the control group. In cases, 29.4% and 70.6% of the subjects had elementary or middle-level education (high school or academic education), while these corresponding values were obtained at 21.2% and 78.8% in controls, respectively (P=0.001). Regarding the occupational status, 20% and 28.8% of the cases and controls were employees, respectively (P<0.001). In total, 39.4% of the cases sleep equal to or more than 2 h during the day; however, this frequency was 27.4% in controls (P<0.012). Furthermore, the frequency of sleep duration less than 6 h at night was significantly higher in cases (25.9%), compared to that in controls (P<0.001) (7.7). Moreover, the family history of depression in the controls (41.8%) was significantly lower (P<0.001) than that in the controls (23.2%).

 Based on HFIAS, the higher score indicates food insecurity; therefore, the cases obtained significantly higher scores (5.58±5.06), compared to the controls (3.49±1.72) (P<0.001).

**Table 1:** Food groups used in the factor analysis

**Food items**	**Subgroups**
1- Refined grains	Lavash bread (Iranian bread), Baguette, Rice, Macaroni
2- Whole grains	Iranian dark bread (Barbari, Sangak, Taftoon), Barley, Bulgur
3- High-fat dairy	High-fat milk, High-fat yogurt, Cream cheese, Cream and butterfat, Ice cream
4- Low-fat dairy	Low-fat milk, Low-fat yogurt, White cheese, Curd, Dough
5- Visceral meat	Heart, Liver, Tongue, Brain, Chest, Pancreas, and Abdomen
6- Red meat	Beef, Lamb, Minced meat
7- Poultry	Chicken
8- Egg	Egg
9- Cruciferous vegetables	Cauliflower, Cabbage
10- Yellow vegetables	Carrots
11- Green leafy vegetables	Spinach, Lettuce
12- Other vegetables	Tomatoes, Cucumber, Eggplant, Onion, Green pea and Beans, Pumpkin, Mushrooms, Red and green peppers, Turnip, Corn and maize, Garlic
13- Potatoes	Boiled and fried potatoes
14- Fruits	Pears, Apricots, Cherries, Apples, Raisins or Grapes, Bananas, Cantaloupe, Watermelon, Oranges, Grapefruit, Kiwi, Strawberries, Peaches, Nectarine, Tangerine, Mulberry, Plums, Persimmons, Pomegranates, Lemons, Pineapples, Fresh Figs, and Dates
15- Legumes	Chickpeas, Lentils, Beans, Peas, Soybeans
16- Non-hydrogenated fats	Vegetable oils (except for olive oil)
17- Hydrogenated fats	All types of Solid oil, Animal oil, Animal butter, Margarine
18- Olive	Olives, Olive oil
19- Nuts	Almonds, Peanuts, Walnuts, Pistachio, Hazelnuts, Seeds
20- Salt	Salt
21- Fast foods	Pizza, Sandwich, Sausage, Hamburger
22- Snacks	Puff, Chips
23- Sweets and desserts	All types of Sweets, Chocolates, Cakes, and muffins
24- Sugars	Sugars, Chocolates, Candies, Gaz (an Iranian confectionery made of sugar, nuts, and tamarisk), Sohan
25- Tea	Tea
26- Coffee	Coffee, Instant coffee
27- Honey or jam	Honey or jams
28- Ketchup	Ketchup
29- Mayonnaise	Mayonnaise
30- Fish	All types of fish
31- Soft drinks	Soft drinks
32- Broth	Broth
33- Pickle	Any Pickle
34- Tuna	All types of Canned fish

Table 3 indicates the factor loading of major dietary patterns using PCA analysis. Additionally, positive loadings demonstrate a positive association with the pattern, while negative loadings reveal an inverse association with it. The PCA identified five major dietary patterns in all participants, namely Healthy, Sugar, and Fast foods, Western, Traditional, and Unhealthy patterns. The “Healthy” pattern included a high amount of yellow vegetables, eggs, nuts, green leafy vegetables, fish, cruciferous vegetables, and olives. The “Sugar and Fast foods” pattern contained a high amount of mayonnaise, pickle, and honey or jam.

**Table 2:** Comparison of the demographic characteristics of all the participants

**Categorical variables**	**Cases (n=170)**		**Controls (n=340)**		
	**Number**	**Percent**	**Number**	**Percent**	***P*** **-value**
**Education Level**				
Elementary	25	14.7	15	4.4	0.001
Middle school	25	14.7	57	16.8	
High school	73	42.9	115	33.8	
Academic	47	27.7	153	45.0	
**Occupational status**				
Housewife	136	80.0	242	71.2	0.001
Employee	11	6.5	71	20.9	
Self-employed	23	13.5	27	7.9	
**Marital status**					
Single	47	27.6	71	20.9	0.046
Married	117	68.8	264	77.6	
Divorced	6	3.5	5	1.5	
**Place of living**					
Urban	153	90.0	319	93.8	0.110
Rural	178	10.0	21	6.2	
**Sleep duration during the day (h)**
0-1	103	60.6	247	72.6	0.012
2-3	52	30.6	76	22.4	
>3	15	8.8	17	5.0	
**Sleep duration at night (h)**
<6	44	25.9	26	7.7	0.001
>6	126	74.1	314	92.3	
**Smoking**					
Yes	4	2.4	0	0.0	0.004
No	165	97.6	340	100	
**Family history of depression**
Yes	71	41.8	79	23.2	0. 001
No	99	58.2	261	76.8	
**Income (Million Rials)**
<10	36	21.2	50	14.7	0.006
10-20	81	47.6	132	38.8	
20-30	38	22.4	93	27.4	
>30	15	8.8	65	19.1	
**Continuous variables**	**Mean**	**SD**	**Mean**	**SD**	***P-*** **value**
Age	36.97	11.28	36.07	10.58	0.374
Body mass index (kg/m^2^)	26.31	5.48	26.59	9.09	0.719
Metabolic equivalent of task	2046.09	1706.23	2685.61	1746.02	0.865
Food security	5.58	5.06	3.49	1.72	0.001

 The “Western” pattern was heavily loaded with sweets, desserts, sugars, snakes, fast food, mayonnaise, and soft drinks. Moreover, the “Traditional” pattern included refined grains, fast food, salt, whole grains, and high-fat dairy. The “Red meat and oils” pattern was heavily loaded with liquid oil, butter, red meat, refined grains, and fruit juice.

The ORs and their 95% CI for depression in five dietary patterns are shown in Table 4. In crude and two adjusted models, the “Healthy pattern” was inversely associated with the odds of depression. The OR in the crude model was (OR: 0.59; 95% CI: 0.46, 0.75; *P*≤0.001). Moreover, the ORs for the adjusted models 1 and 2 were (OR: 0.56; 95% CI: 0.43, 0.73; *P*≤0.001) and (OR: 0.61; 95% CI: 0.46, 0.81; *P*≤0.001), respectively. In crude and two adjusted models, the “Western pattern” had a significant positive association with the odds of depression. Therefore, the OR in the last model was (OR: 1.29; 95% CI: 1.06, 1.59; *P*=0.011). The “Traditional pattern” increased the odds of depression; however, this difference was not statistically significant in the adjusted last model (OR: 1.16; 95% CI: 0.94, 1.43; *P*=0.144). On the other hand, there was a significant positive relationship between “Traditional pattern” and depression in the crude model (OR: 1.25; 95% CI: 1.04, 1.50; *P*=0.016) and adjusted model 1 (OR: 1.25; 95% CI: 1.03, 1.53; *P*=0. 004). It is noteworthy to mention that the association of “Sugar and fast food” and “red meat and oils” patterns with depression was not significant in any crude and adjusted models.

**Table 3:** Factor loading^a^ of major dietary patterns in all participants

**Food groups**	**Dietary patterns**				
	**Healthy**	**Sugar and fast food**	**Western**	**Traditional**	**Red meat and oils**
Refined grains	-^b^	-	-	0.603	0.324
Whole grains	0.245	-	-	0.378	-
Low-fat dairy	-	0.657	-	-	-
High-fat dairy	-	-	-	0.598	-
Green leafy vegetables	0.540	-	-	-	-
Yellow vegetables	0.649	-	-	-	-
Cruciferous vegetables	0.437	-	-	-	0.200
Other vegetables	0.388	0.269	-	--	-
Fruits	0.500	0.406	-	-	-
Legumes	-	-	-	0.488	-
Nuts	0.553	0.287		-	
Red meat	-	-	0.340	-	0.343
Poultry	-	0.437		-	-
Egg	0.591	-	-	0.231	-
Potatoes	-	-	-	-	0.277
Fish	0.444	-	-	-	0.233
Fast food	-	-	0.521	0.373	-
Visceral meat	0.243	-	-	0.285	0.220
Butter	-	-	-	-	0.343
Fruit juice	-	0.375	0.217	0.213	0.611
Olive	0.490	-	-	-	-
Solid Oil	-	-	0.276	0.255	-
Liquid Oil	-	0.276	-	-0.227	0.563
Honey or jam	-	0.655	-	-	-
Sweets and desserts	-	-	0.664	-	-
Sugars	-	0.401	0.642	-	-
Snacks	-	-	0.562	-	-0.201
Soft drinks	-	-	0.485	-0.212	0.331
Tea and coffee	-	-	-	-	-0.379
Salt	-	0.316	-	0.395	-
Ketchup	-	-	0.511	-0.225	-
Pickle	-	0.576	-	-	-
Mayonnaise	-	0.488	0.447	-0.253	0.326
Broth	-	-	-	-	0.259

^a^ Extraction Method: Principal Component Analysis, Rotation Method: Varimax with Kaiser Normalization. ^b^ Blank represents Factor loadings < 0.2 for simplicity. Kaiser-Meyer-Olkin measure of sampling adequacy: 0.63

**Table 4:** Odds Ratio and 95% confidence interval of depression for dietary pattern scores using logistic regression analysis

**Dietary patterns**	**Crude model**		**Model 1**		**Model 2**	
	**OR (95% CI)**	**P-value**	**OR (95% CI)**	**P-value**	**OR (95% CI)**	**P-value**
Healthy	0.59 (0.46, 0.75)	0.001	0.56 (0.43, 0.73)	0.001	0.61 (0.46, 0.81)	0.001
Sugar and fast food	1.05 (0.87, 1.26)	0.580	1.04 (0.85, 1.26)	0.690	0.98 (0.79, 1.21)	0.892
Western	1.21 (1.01, 1.45)	0.033	1.31 (1.08, 1.59)	0.006	1.29 (1.06, 1.59)	0.011
Traditional	1.25 (1.04, 1.50)	0.016	1.25 (1.03,1.53)	0.024	1.16 (0.94, 1.43)	0.144
Red meat and oils	0.91 (0.75, 1.10)	0.335	0.93 (0.76, 1.14)	0.494	0.94 (0.76, 1.16)	0.598

Model 1 is adjusted for marital status, education level, family history of depression, occupation, smoking, daily sleep, and night sleep. Model 2 is adjusted for Model 1 in addition to income and food security.

## Discussion

In the present study, five dietary patterns were identified using the PCA. Among these five patterns, “Healthy”, “Western”, and “Traditional” patterns showed a significant relationship with depression. Therefore, the “Western” and “Traditional” dietary patterns were positively associated with depression, while adherence to the “Healthy” pattern reduced the depression risk by 39% in the last adjusted model. Moreover, the adherence to “Western” and “Traditional” patterns increased the risk of depression by 29% and 16% in the last model, respectively. The dietary patterns obtained using PCA were similar to the extracted patterns in the previous studies ^5,14^. The variance explained by three dietary patterns (Healthy: 8.21%, Western 7.47%, and Traditional: 6.22%) was similar to that obtained in other studies using the PCA ^6,27^.

 The present study demonstrated that “Western” dietary and “Traditional” patterns, as the unhealthy dietary patterns, were characterized by a higher intake of sweets and dessert, red meat, fast food, mayonnaise, ketchup, snacks, refined grains, high-fat dairy, salt, legumes, and soft drinks.

This study showed a positive association between unhealthy dietary patterns (Western dietary and Traditional patterns) and the risk of depressive symptoms. Our findings were consistent with some of the previously conducted epidemiological studies throughout the world that have reported a significant association between unhealthy dietary pattern and depression risk ^5,28-33^. The positive relationship between an unhealthy dietary pattern and depression can be justified in this way. Available foods, such as sugar, red meat, low levels of natural antioxidants, fiber, and omega-3 fatty acids in this pattern, activate inflammatory pathways. High consumption of processed and red meats was associated with higher levels of low-grade inflammation (C-reactive protein) and subsequent brain atrophy, which is in turn positively associated with depression ^34^. Moreover, high intakes of sugar, fast foods, snakes, and soft drinks in the Western pattern were associated with depression due to the altered endorphin levels and oxidative stress^33^.

A high level of inflammatory markers exists in depressed patients. Some studies have revealed that an inadequate diet is one of the factors that increase the inflammatory markers in individuals. Microglia begin to produce cytokines (e.g., INF, IL2, and IL1) ^35^during illness or damage. These factors stimulate the pathway of tryptophan to kynurenic acid (N-methyl-D-aspartic acid [NMDA] antagonist) and kyolinic acid (NMDA agonist) by indoleamine dioxygenase. Subsequently, the levels of tryptophan and serotonin decreased based on this mechanism ^36^.

Moreover, pro-inflammatory cytokines may be effective in the development or progression of depressive disorders by inhibiting the expression of brain-derived neurotrophic factor, which interferes with the metabolism of neurotransmitters and reduces the serotonin precursors, such as tryptophan ^37^. Additionally, high consumption of red and processed meats (containing large amounts of saturated fatty acid) was associated with higher levels of low-grade inflammation (C-reactive protein) and subsequent brain atrophy that are positively associated with depression ^34^. Cerebral neurotrophic factor plays an effective role in depressive disorder through oxidative protection of neurons and stimulation of neurogenesis ^38^.

In this study, healthy dietary pattern included vegetables, fruits, eggs, nuts, olive oil, fish, and whole grains rich in these nutrients. The results of this study were consistent with the findings of previously conducted studies ^39-42^. In the present study, there was a significant inverse relationship between adherence to the healthy pattern and the risk of depression. This relationship remained significant even after adjusting for confounding variables, such as income, education level, family history of depression, occupation, smoking, daily sleep, night sleep, and food security. A healthy diet pattern has reduced the probability of depression by several possible mechanisms.

This diet includes tryptophan (precursor of serotonin) and is replete with omega 3 and omega 6 fatty acids that have a role in anti-inflammatory pathways ^40^. Furthermore, the presence of antioxidant compounds (e.g., Vitamins C and E) in fruits and vegetables may reduce the neuronal damage induced by oxidative stress, which is believed to decrease depression^41,42^. Similarly, fruits, vegetables, and whole grains are rich sources of dietary fiber and folate, which have beneficial protective effects against depression. High fiber intakes are inversely associated with insulin resistance, which is the risk factor for depression^43^. Additionally, the level of folic acid is high in this pattern, and some studies have shown that folate deficiencies may result in the increased homocysteine concentrations and reduced S-adenosylmethionine, which have a fundamental role in neurotransmitters^44^.

The protective effect of fish consumption may be attributed to its high content of long-chain omega-3 polyunsaturated fatty acids contributing to the brain functioning and serotonin neurotransmission^45^. The results of studies have shown that adherence to the “Western” dietary pattern was positively associated with low-density lipoprotein cholesterol (LDL-C) level among the study population ^46^. Furthermore, the findings showed that patients with major depressive disorders had an altered lipid profile, compared to the controls. In this regard, LDL and omega-6 levels were raised, while a decrease was observed in high-density lipoproteins and omega-3 levels ^47^. In depressed patients, omega-3 long-chain polyunsaturated fatty acids produced anti-inflammatory eicosanoids that reduced levels of pro-inflammatory cytokines^14,48^. This is characterized by the relationship between higher levels of fish consumption in healthy dietary patterns and decreased incidence of depression.

 The strengths of the present case-control study include the utilization of regression models adjusted for possible confounding variables. Additionally, trained dieticians collected dietary data, and food consumption was assessed through a validated-FFQ questionnaire. Moreover, the dietary patterns were derived from the PCA method. Regarding the limitations of the present study, one can refer to the probability of information bias and recall bias. In addition, even though confounding factors have been adjusted in the analysis, it was not possible to ignore the potential effect of other unmeasured factors, such as cultural factors.

## Conclusions

 Based on the results, it seems that unhealthy dietary patterns were positively associated with depression risk, while the healthy dietary pattern is associated with a reduced risk of depressive symptoms. Present findings provide further insight into a better understanding of the associations between dietary patterns and depressive symptoms. Future studies and trials are required to elucidate whether a true causal association exists or not.

## Acknowledgements

 The authors wish to thank the participants for their cooperation in the study.

## Conflict of interest

 There is no conflict of interest regarding the publication of this study.

## Funding

 This study was supported by Urmia University of Medical Sciences, Urmia, Iran.

## Highlights

- Western and traditional dietary patterns increase the risk of depression in adult females.
- Adherence to healthy diets has been associated with better mental health.
- Depression is associated with altered eating patterns in many, but not all the cases.
